# Fluid or Fuel? The Context of Consuming a Beverage Is Important for Satiety

**DOI:** 10.1371/journal.pone.0100406

**Published:** 2014-06-19

**Authors:** Keri McCrickerd, Lucy Chambers, Martin R. Yeomans

**Affiliations:** School of Psychology, University of Sussex, Brighton, United Kingdom; INRA, France

## Abstract

Energy-containing beverages have a weak effect on satiety, limited by their fluid characteristics and perhaps because they are not considered ‘food’. This study investigated whether the context of consuming a beverage can influence the satiating power of its nutrients. Eighty participants consumed a lower- (LE, 75 kcal) and higher-energy (HE, 272 kcal) version of a beverage (covertly manipulated within-groups) on two test days, in one of four beverage contexts (between-groups): thin versions of the test-drinks were consumed as a thirst-quenching drink (*n* = 20), a filling snack (*n* = 20), or without additional information (*n* = 20). A fourth group consumed subtly thicker versions of the beverages without additional information (*n* = 20). Lunch intake 60 minutes later depended on the beverage context and energy content (*p* = 0.030): participants who consumed the thin beverages without additional information ate a similar amount of lunch after the LE and HE versions (LE = 475 kcal, HE = 464 kcal; *p* = 0.690) as did those participants who believed the beverages were designed to quench-thirst (LE = 442 kcal, HE = 402 kcal; *p* = 0.213), despite consuming an additional 197 kcal in the HE beverage. Consuming the beverage as a filling snack led participants to consume less at lunch after the HE beverage compared to the LE version (LE = 506 kcal, HE = 437 kcal; *p* = 0.025). This effect was also seen when the beverages were subtly thicker, with participants in this group displaying the largest response to the beverage’s energy content, consuming less at lunch after the HE version (LE = 552 kcal, HE = 415 kcal; *p*<0.001). These data indicate that beliefs about the consequences of consuming a beverage can affect the impact of its nutrients on appetite regulation and provide further evidence that a beverage’s sensory characteristics can limit its satiating power.

## Introduction

Overweight and obesity have increased worldwide [1] reflecting overconsumption relative to energetic need. This has led researchers to question whether the satiety value of foods (the extent to which a food suppresses hunger and future food intake once it has been consumed) can be improved to promote better energy regulation [2]. Regular ingestion of energy in beverages is thought to contribute to excessive energy intake and weight gain [3]–[6] because fluid calories have been shown to have a weak effect on satiety [7]–[10], and governments across the world are considering the ways in which population-wide consumption of these products can be reduced [11]–[13]. Yet beverage products are increasingly popular, with leading producers reporting record global sales in the last 10 years [14]: in the UK energy from beverages now contributes to almost a fifth of an adult’s daily energy intake [15]. Therefore it is important to find ways to improve the satiating power of energy-containing beverages.

The development of satiety integrates early cognitive and sensory signals from a food with later post-ingestive nutrient effects [16], [17]. So what features of a beverage limit its satiating power and can these be changed? Research has shown that a beverage’s sensory characteristics are important: beverages often fail to suppress hunger and future energy intake compared to equi-caloric solid and semi-solid versions of the same food [7]–[10], [18]. For example, energy consumed as apple juice was less satiating than the same nutrients consumed as apple puree, which was in-turn less satiating than apple slices [8]. This could be because liquids are consumed faster than more viscous food forms which reduces the duration of oro-sensory exposure [19]–[21]. A low viscosity but high-energy beverage requires little oro-sensory processing and this might limit its anticipated satiating effect [22], [23] and elicit inadequate anticipatory physiological responses (such as cephalic phase salivation and gut-peptide release), which together might weaken the satiating effect of the nutrients it contains [7], [24]. Indeed, recent research from our laboratory suggests that the actual satiating power of a higher-energy beverage depended on its sensory context [25]–[28]. When participants consumed flavour-matched higher- and lower-energy versions of a thin beverage mid-morning they felt equally full and consumed similar amounts at lunch after both drinks, despite consuming 200 kcal extra in the higher-energy version. But when the two versions of the beverage were made to taste subtly thicker and creamier (without adding any extra energy) participants felt fuller and ate significantly less at lunchtime after they consumed the higher-energy version. Importantly, a reduction in lunch intake was not seen after the sensory-enhanced lower-energy beverage, indicating that this was not a general effect of enhanced sensory context on satiety, but a sensory-nutrient interaction where thick and creamy sensory cues only improved satiety when they predicted the delivery of nutrients. Thus, nutritive beverages may have a weak effect on satiety responses if they lack appropriate sensory cues signalling the delivery of nutrients.

Energy-containing beverages may also have a weak effect on satiety if they are not consumed in the context of ‘food’. For example, presenting a liquid as a soup suppressed hunger more than the same liquid consumed as a beverage [10], [18], [29]. Whilst ‘eating’ a liquid with a spoon might influence satiety by increasing oro-sensory exposure time during consumption [30] this may also heighten beliefs that a food is being consumed, compared to drinking the liquid which may be associated more with thirst [31]. On the other hand, meal-replacement ‘shakes’ are drank like a beverage but marketed and consumed as a ‘meal’ rather than as a ‘drink’, and when consumed in this context have been shown to promote weight loss [32]. Indeed, experimental studies indicate that satiety-related beliefs are important for appetite control [33]–[35]. For example, participants ate more at a test meal after consuming a food perceived to be a *snack* compared to participants who consumed the same food but believed it to be a *meal* [33]. This may be because a meal is associated with greater satiety and so foods consumed in this context are expected to be more satiating than the same foods consumed as a snack. Importantly, beliefs about the satiating effects of food can influence the actual experience of satiety: in one study participants reported feeling more full and less hungry after consuming the same smoothie believed to contain a large compared to a small portion of fruit [36] whilst in another study consuming a liquid with the expectation that it would solidify in the stomach (but that actually remained a liquid) elicited slower gastric-emptying and enhanced the experience of satiety [7]. With these previous findings in mind, an energy-containing beverage consumed in the context of a snack might be expected to be more satiating than the same beverage consumed in a less satiety-relevant context, such as a drink. Generating beliefs of this kind might be one way to influence the satiating power of nutrients consumed as a beverage without the need to modify its sensory characteristics, which could be unacceptable to consumers.

To test this idea, participants in this study consumed a higher- and lower-energy version of a fruit-juice based beverage presented in one of four contexts varying in textural and cognitive cues: *thin texture* with no additional context information; thin texture presented as a new “*thirst-quenching beverage*”; thin texture presented as a new “*filling snack*”; *thick texture* with no additional information. The subtly thicker versions were intended as a positive control to detect the sensory-enhanced satiety reported in our previous findings [25]–[28], allowing for the comparison between changing satiety-relevant beliefs and the alternative approach of modifying textural cues to influence sensitivity to nutrients consumed in a beverage. The beverage’s energy content was covertly manipulated. It was predicted that participants who consumed the thin versions of the beverage with either no information or in the context of it being a thirst-quenching drink would not respond to the covert energy difference between the beverages by adjusting their intake at a later lunch-time meal, while those who received the beverage presented as a filling snack or with added satiety-relevant sensory cues would adjust their lunch intake depending on the beverages energy content.

## Materials and Methods

### Ethics Statement

This research was approved by the University of Sussex Life Science Research Ethics Board and all participants gave written informed consent to take part.

### Design

A two-factor 4×2 mixed design was used to assess the satiety value (as measured by changes in rated appetite and intake at a later meal) of a beverage presented in one of four cognitive and sensory *beverage contexts* (measured between-groups: thin/no information; thin/thirst-quenching: thin/filling; thick/no information) varying in *energy content* (measured within-groups: lower-energy (LE) vs. higher-energy (HE)). Our previous research identified a large interactive effect of beverage energy and sensory context on later intake [28] (power = 0.85 to detect effect size *f* = 0.53). Based on this, a sample size calculation for a mixed ANOVA design, where the effect of the cognitive manipulation was unknown but assumed to be smaller (effect size *f* = 0.25, power = 0.95) suggested 64 participants for the study (*n* = 16 in each group) which was increased to 20 per group (*n* = 80) to allow for counterbalancing and any exclusions.

### Participants

Eighty female participants were recruited to take part in a study investigating ‘Food and Mood’ from a volunteer database held by the University of Sussex Ingestive Behaviour Unit (SIBU). Eligible participants were non-smokers, not diagnosed with an eating disorder, without allergies or aversions to any of the test food ingredients and not taking prescription medication or currently dieting. Participants did not have a restrained eating style as measured by the Three Factor Eating Questionnaire (TFEQ) [37]. Participants were randomly assigned to one of the four beverage context groups, which did not statistically differ in mean age (years), BMI (kgm^−2^), TFEQ-Restraint score (representing the tendency to restrict food intake) and TFEQ-Disinhibition score (representing the tendency to overeat) (see Table 1).

**Table 1:** Mean (± SD) Age, BMI, TFEQ restraint and TFEQ disinhibition scores for the participants in the different beverage context groups.

	Thin	Thin	Thin	Thick	*p*-value*
	No information	Thirst-quenching	Filling	No information	
Age (years)	20.5±2.4	20.2±2.3	19.5±2.0	20.5±5.4	0.809
BMI (kgm^−2^)	22.3±2.8	22.9±2.5	22.2±2.4	22.6±3.6	0.850
TFEQ-R	3.1±1.7	3.4±2.0	3.1±2.0	3.3±1.9	0.949
TFEQ-D	6.0±3.4	6.2±2.3	6.3±3.8	6.5±3.3	0.958

*The *p*-value from a one-way between-groups ANOVA comparing each of the demographic measures across the 4 test-groups.

### Test Foods and Drink

On each test day all participants consumed a standard breakfast in the lab, followed by the test drink and later an *ad libitum* lunch. They received a 500 ml bottle of spring water (Sainsbury’s, UK) to drink in between these sessions. Breakfast consisted of cereal (“Crunchy Nut Cornflakes”, Kelloggs, UK: 60 g), semi-skimmed milk (Sainsbury’s, UK: 160 g) and orange juice (Sainsbury’s, UK: 200 g), which provided 440 kcal. Participants also consumed an *ad libitum* lunch in the lab, served in 450 g portions consisting of 250 g cooked conchiglie pasta combined with 200 g fresh tomato and basil pasta sauce (both Sainsbury’s, UK). Each portion contained 544 kcal.

**Figure 1:**
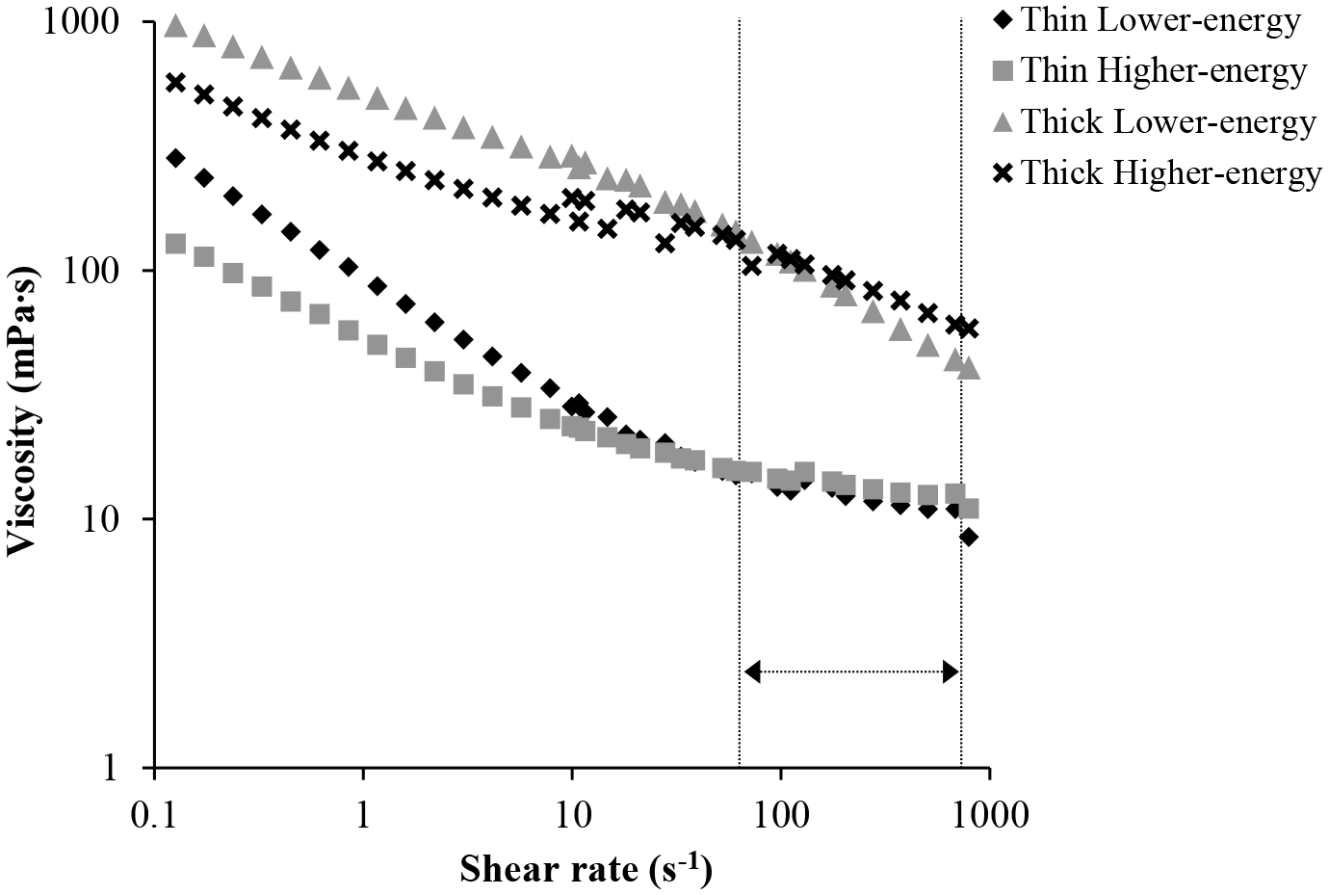
Viscosity of the four test drinks under shear. The section marked with an arrow represents viscosity measured between shear rates 80–700 s^−1^, which are thought to best represent speeds associated with the perceived viscosity of fluids [38].

The four test drinks were developed in-house based on a recipe described in a previous study [23] using commercially available ingredients. A higher-energy (HE) and lower-energy (LE) version of a thin and thick drink were prepared as a 320 g portion, each containing fresh mango, peach and papaya fruit juice (LE and HE = 100 g; Tropicana Products, Inc.), 0.1% fat fromage frais (LE = 55 g, HE = 30 g; Sainsbury’s UK), water (LE = 130 g, HE = 100 g;) and peach flavoured diluting drink (LE and HE = 11 g; ‘Robinsons’ from Britvic, UK). The HE versions of the drink also contained 55 g of maltodextrin (Cargill, UK) such that one portion of the HE drink contained 272 kcal while the LE version contained 75 kcal. A small quantity of aspartame (0.03 g: Ajinomoto, Japan) was added to the LE drinks to match sweetness to the HE versions. Tara gum (Kalys Gastronomie, FR) was used to subtly increase the viscosity of the thick drinks and to match for the slight increase in viscosity caused by the addition of maltodextrin to the HE versions (thin LE = 0.2 g; thin HE = 0.0 g; thick LE = 1.2 g; thick HE = 1.0 g). Rheological measurements were conducted at 5C on a Bohlin Rotational Rheometer at shear rates 0.1–800 s^−1^ using parallel plate geometry (60 mm diameter) and a gap size of 1.0 mm (Malvern Instruments Ltd.). Perceived thickness of a fluid containing a similar polysaccharide thickener (guar gum) was reported to most strongly correlate with viscosity measured at shear rates of ≈80–700 s^−1^ [38] and at these speeds the thicker drinks were more viscous than the thin versions and the high and low energy drinks were well matched, see Figure 1. Colour was matched between all the drink samples by small additions of natural food colouring. In our previous studies participants rated the drinks to be equally pleasant and sweet, the thicker drinks as significantly thicker and creamier than the thin versions, and were unaware of the energy manipulation [23], [28].

### Beverage Context

Participants consumed higher- and lower-energy versions of the beverage in one of the four drink context conditions. One group consumed thin versions of the beverage with no additional information (*thin/no information* group). Two more groups consumed the same thin beverages but with some additional contextual information and were informed during the first session that they would consume a new product that had been designed by a food and drink company. The *thin/thirst-quenching* group were told that they would be trying a new drink product designed to affect feelings of thirst, whereas participants in the *thin*/*filling* condition were told they were trying a new snack, which would affect feelings of hunger and fullness. The final group consumed thicker versions of the beverage without any additional information (*thick/no information* group). All participants were informed that they would consume the *drink/snack* and evaluate it alongside their mood. In the two information groups participants were also presented with an information sheet “from the manufacturer” to standardise the information they received about the beverages, see Table 2. All the drinks were presented and consumed from a clear, pre-sealed plastic bottle using a straw.

**Table 2:** A description of the information provided to participants in the *thin/thirst-quenching* and *thin/filling* beverage context groups.

A refreshing drink to quench your thirst	A filling snack to keep hunger away
This is a drink that has been developed to stop youfrom feeling thirsty and to keep you hydratedthroughout the day	This is a snack that has been developed to stop you from feeling hungry and to keep you full throughout the day
Drinking enough is an important part of our dietwhich helps our body to work properly through theday. When you don’t drink enough you can becomedehydrated and this can affect how you feel.	Eating enough is an important part of our diet which helps our body to work properly through the day. When you don’t snack on the correct foods you can become hungry and this can affect how you feel.
If you are dehydrated you might start to feel thirsty	If you have not eaten enough, you might start to feel hungry

### Procedure

**Figure 2:**
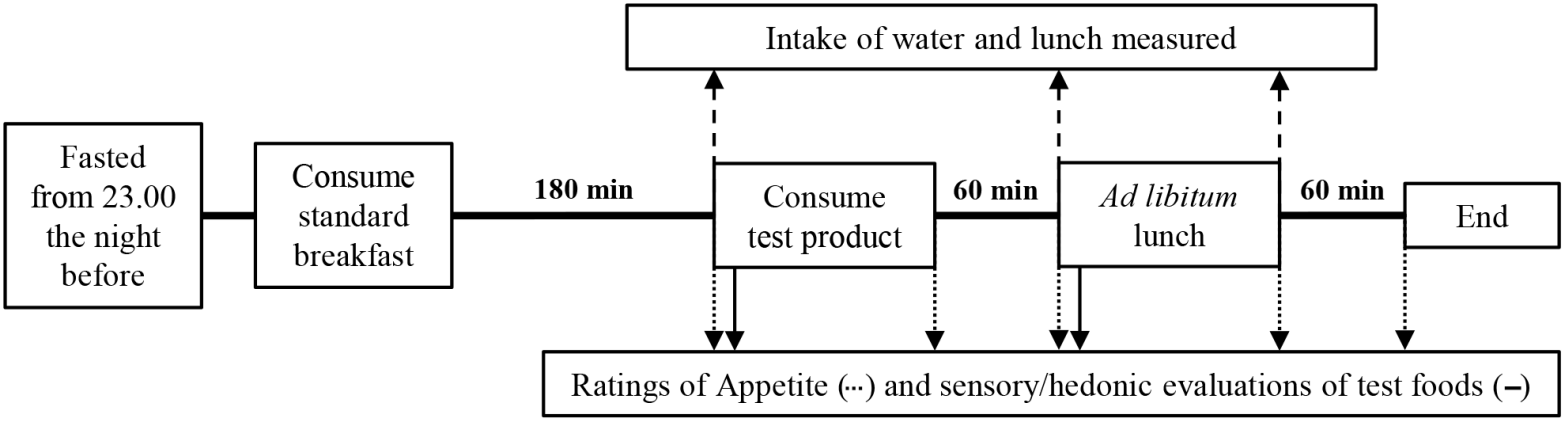
Schematic summary of the test day procedure.

Figure 2 summarises the main procedure and measurement points throughout the test days. Participants attended the SIBU on two non-consecutive days, arriving for breakfast at a scheduled time between 8∶30 and 10∶30 having consumed nothing but water from 23∶00 the evening before. Once they had consumed all of their breakfast, participants left the laboratory for three hours and were instructed to consume only water in this time. They were given a 500 ml bottle of water to take away and instructed to drink from this if needed and to bring the bottle back for the next session when it would be topped up. Water intake was covertly measured.

After three hours participants were shown to a testing cubicle with a PC computer running the Sussex Ingestion Pattern Monitor software (SIPM: University of Sussex) [39]. To begin this part of the session, participants completed a set of appetite ratings called “Mood Questions” (pre-drink appetite). They were asked “How <target> do you feel right now?” and instructed to indicate the extent to which they felt *hungry*, *full* and *thirsty* by placing a marker along a 100 mm Visual Analogue Scale (VAS). The scale response ranged from “Not at all <target>” (0) to “Extremely <target>” (100) and these ratings were embedded amongst distracter “mood” ratings of *tired*, *happy*, *headachy*, *anxious*, *energetic*, *nauseous* and *alert*. All VAS ratings were presented in a randomised order and only the appetite ratings were analysed. Having completed these ratings all participants received the test-drink and, depending on their beverage context condition, they were given additional information regarding their “*drink*” or “*snack*” product. All participants were then instructed to taste the product using the straw provided and evaluate how *thick*, *creamy*, *pleasant*, *sweet* and *familiar* it was, using the same randomised VAS format as the appetite ratings. They were then asked to consume all of the drink/snack and complete a second set of “mood” questions (post-drink appetite ratings). Once they had finished participants received their refilled bottle of water and were asked not to consume anything but water while they waited for their lunch session.

Returning to a test cubicle 60 minutes later, participants began lunch by handing in their water bottle to be topped-up and completing the third set of “mood” questions (60 minute appetite). The 60 minute time gap was based on unpublished pilot data investigating the effects of the test drink’s energy content on changes in rated appetite over 120 minutes post-consumption (*n* = 49), which indicated an effect of beverage energy content on subjective appetite from 60 minutes onwards in a similar participant population (i.e. non-dieting females reporting low dietary restraint). The time frame of a cognitive effect was unknown, but previous research has indicated that satiety-relevant beliefs can influence rated appetite 15–240 minutes after consumption and intake of another meal after 240 minutes [7]. Lunch intake was measured using a concealed balance (Sartorius model BP4200) linked to the SIPM, which was secured under a placemat and covertly measured and recorded lunch intake. At the beginning of the lunch phase participants were presented with a sample of their pasta lunch and prompted to taste and rate how *familiar*, *pleasant* and *salty* it was and then asked again to rate how *hungry*, *full* and *thirsty* they felt (pre-lunch appetite). Next, they were given a 450 g serving of the pasta lunch that was placed on the placemat and both the experimenter and on-screen instructions explained that they could eat as much as they liked and would receive refills when needed. After 350 g had been consumed an alert sounded and they were instructed that a refill was required, at which point the researcher presented another 450 g serving of pasta. Participants could end the consumption phase by selecting ‘meal terminated’ when ready, unless they were at a refill stage in which instance they would have to receive their refill first. This was to limit using the refill as a reason to end the meal. The refill procedure also prevented participants from completely finishing the portion in the bowl, another strong external cue for meal termination. Participants completed a final set of mood questions (post-lunch appetite) to end the lunch session. Participants were asked to not eat or drink anything but water for another hour after lunch in order to limit the potential for the future availability of food to influence lunch intake decisions. They completed a paper version of the mood questions at the end of this hour that was returned at the start of the next session but these data are not reported.

Overall participants completed two test days that were identical except for the energy content of the test beverage. Participants received the LE beverage in one session and the HE version in the other, the order of which was counter-balanced within the four beverage context groups. At the end of the second day participants completed a debriefing questionnaire, after which the purpose of the study was explained and participants were asked to keep this information confidential. Height and weight measurements were recorded and participants had the opportunity to ask any questions before being thanked and receiving £20 for taking part.

### Debrief Questionnaire

The debriefing questionnaire was used to check that all participants were naive to the true purpose of the study and to determine whether those given extra information believed the drinks were designed to be filling/thirst-quenching products. This short questionnaire first asked participants to comment on the purpose of the study (question 1) and then to identify whether they expected the products they consumed to be ‘thirst-quenching’, ‘filling’, ‘both’, ‘neither’ or ‘other’ and to give a reason for their answer (question 2). This was followed by a short series of other questions about their experience of consuming each of the test-foods over the two days (e.g. “Did you think the breakfast/drink product/lunch you consumed was the same on each day? If not, why?”). All questions required a yes/no/unsure answer and an explanation where necessary. Once this sheet was complete participants were verbally debriefed. Participants in the filling/thirst-quenching beverage context groups were then asked whether they believed that a food company had developed the drink/snack they received and their response was noted. It was assumed that participants believed the cognitive manipulations if they a) reported that they expected the drink to be thirst-quenching/filling (in line with their condition) in response to questions 1 and 2 of the debrief sheet and b) indicated that they believed they had consumed a new product from a food company. In-line with these criteria data from four participants were excluded from the final analyses.

### Data Analysis

Since the main aim of the study was to assess the extent to which satiety generated by energy consumed in a drink depended on the cognitive and sensory context in which it was presented, a series of mixed-ANOVAs were used to test the effect of the *beverage context* (between-groups: *thin/no information* vs. *thin/thirst-quenching* vs. *thin/filling* vs. *thick/no information*) and *energy content* manipulation (within-groups: *LE* vs. *HE*) on the key outcome measures of total lunch intake (kcal) and changes in rated appetite, with *rating time* (within-groups: *pre-drink*, *post-drink*, *60 min later*, *pre-lunch* and *post-lunch*) as an additional factor to these analyses. For the lunch intake values, the difference in lunch intake after the HE compared to the LE beverage was calculated as a percentage of the 197 kcal difference between the HE and LE versions. This describes the degree to which participants responded to the additional energy in the HE beverage (197 kcal). A similar ANOVA design was used to analyse the additional variables of water intake throughout the sessions (g) and the sensory and hedonic evaluations of the test foods. The order in which the beverages were consumed (between groups: *LE-HE* vs. *HE-LE*) was initially included in all analyses but this had no significant effect on the main outcomes and was removed from the final analyses. All follow-up analyses used to interpret, where necessary, the direction of any main effects and interactions between the energy content and beverage context report Bonferroni adjusted *p*-values to account for multiple pairwise comparisons performed. When the assumption of sphericity was violated (within-group variable only) the appropriate Greenhouse-Geisser (*ε*<0.75) or Huynd-Feldt (*ε*>0.75) corrected degrees of freedom and *p*-values are reported. Means and SEM are presented throughout results and in figures and tables. Partial eta squared values (*η_p_^2^*) are reported as a measure of effect size for all the main analyses, and indicate the portion of the variance in the outcome measures accounted for by the independent variable(s) (a smaller value indicates a smaller amount of variance). As a general guide *η_p_^2^≥*0.14 represents a large effect, *η_p_^2^≥*0.06 a medium effect, *η_p_^2^≥*0.01 a small effect and *η_p_^2^≤*0.01 is a negligible effect [40]. During the debrief two participants reported controlling their lunch intake (one was following a diet to gain weight and another reported restricting intake) and their data were excluded in addition to the four participants removed because they did not believe the information manipulation. Therefore data from 74 participants was included in the final analyses (thin/no-information, *n* = 19; thin/thirst-quenching, *n* = 17; thin/filling, *n* = 19; thick/no-information, *n* = 19). The outcome of the main findings reported in this manuscript were not affected by including data from those participants who were excluded based on their belief in the cognitive manipulation.

## Results

### Lunch Intake

**Figure 3:**
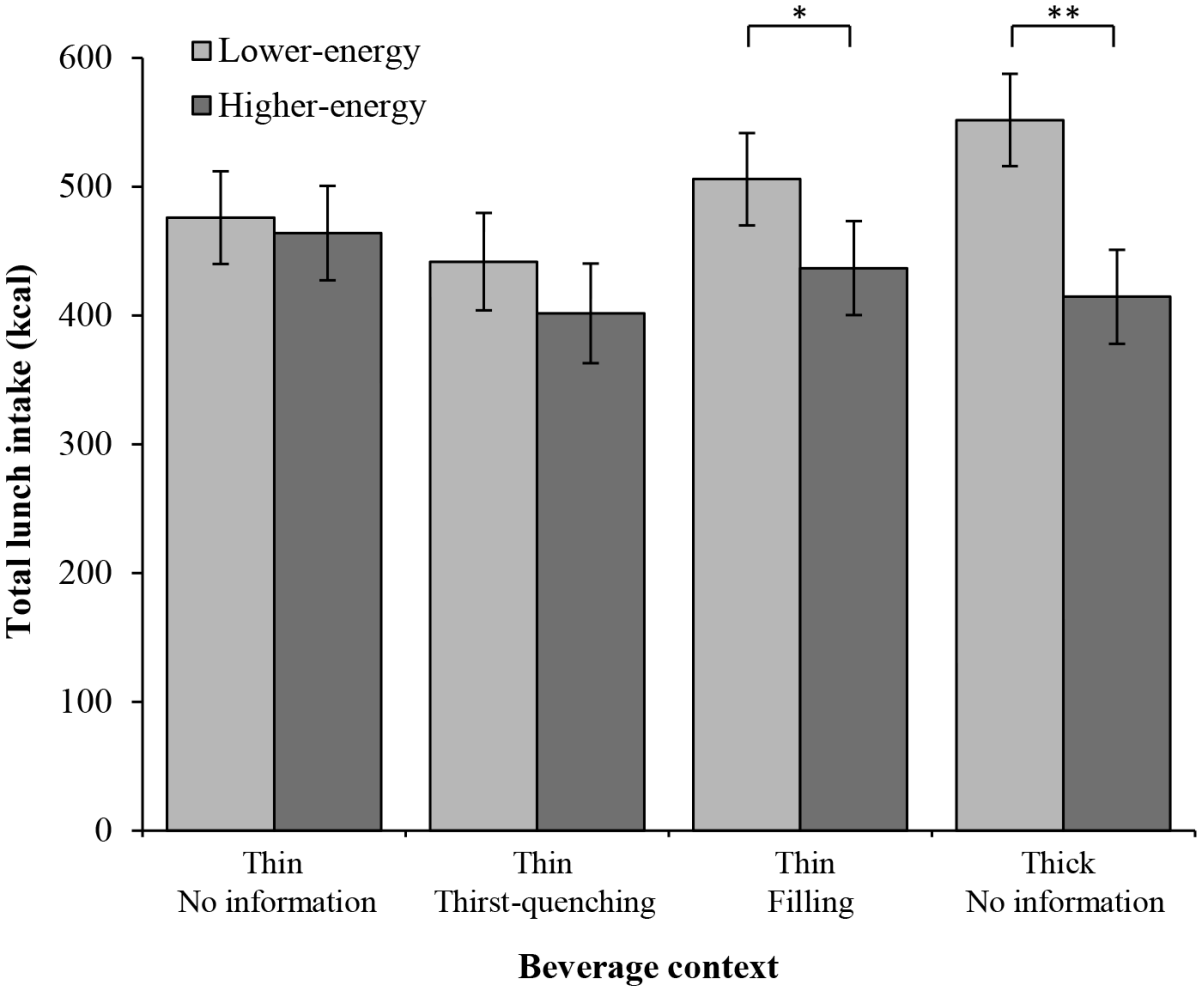
Mean lunch intake (± SEM) after consuming both the lower-energy and higher-energy versions of the drinks across each group, *indicates a significant difference where *p*>0.05 and **p>0.001.

Participants consumed significantly less of the pasta lunch overall after having the HE drink compared to the LE version (*M_HE_* = 429.2±18.6 kcal, *M_LE_* = 493.8±18.2 kcal: *F*(1, 70) = 17.82, *p*<0.001, *η_p_^2^* = 0.20). There was no overall effect of the beverage’s context on lunch intake (*F*(3, 70) = 0.63, *p* = 0.598, *η_p_^2^* = 0.03) but this did interact with energy content to influence lunch intake (*F*(3, 70) = 3.15, *p* = 0.03, *η_p_^2^* = 0.12; see Figure 3). Looking at the effect of energy content within each beverage context, those who consumed the thin beverage with no additional information (*thin/no-information*) consumed a similar amount of lunch after the HE and LE versions (*F*(1, 70) = 0.16, *p* = 0.690) despite consuming almost 200 extra kcal in the HE drink. Similarly, those participants who consumed the thin drink and believed it to be thirst-quenching (*thin/thirst-quenching*) did not significantly differ in the amount they consumed after the HE and LE drink (*F*(1, 70) = 1.58, *p* = 0.213). In contrast, participants who consumed the drink in the context of a snack (*thin/filling*) consumed significantly less after the HE drink compared to the LE version (*F*(1, 70) = 5.25, *p* = 0.025). The largest difference in lunch intake after the HE drink compared to the LE version was seen in the *thick/no-information* group who consumed the beverage in the thick sensory context (*F*(1, 70) = 20.69, *p*<0.001).

The difference in lunch intake after the LE compared to the HE beverage was described as percentage of the additional 197 kcal consumed in the HE version. This indicated that the difference in lunch intake after the LE compared to HE beverage for the *thin/no-information* group equated to 6% of the additional energy in the HE version. In the *thin/thirst-quenching* and *thin/filling* groups this increased to 20% and 35% of the additional energy respectively, while participants in the *thick/no-information* group responded the most, showing a difference in lunch intake that accounted for 70% of the extra energy consumed.

### Changes in Rating Appetite

**Figure 4:**
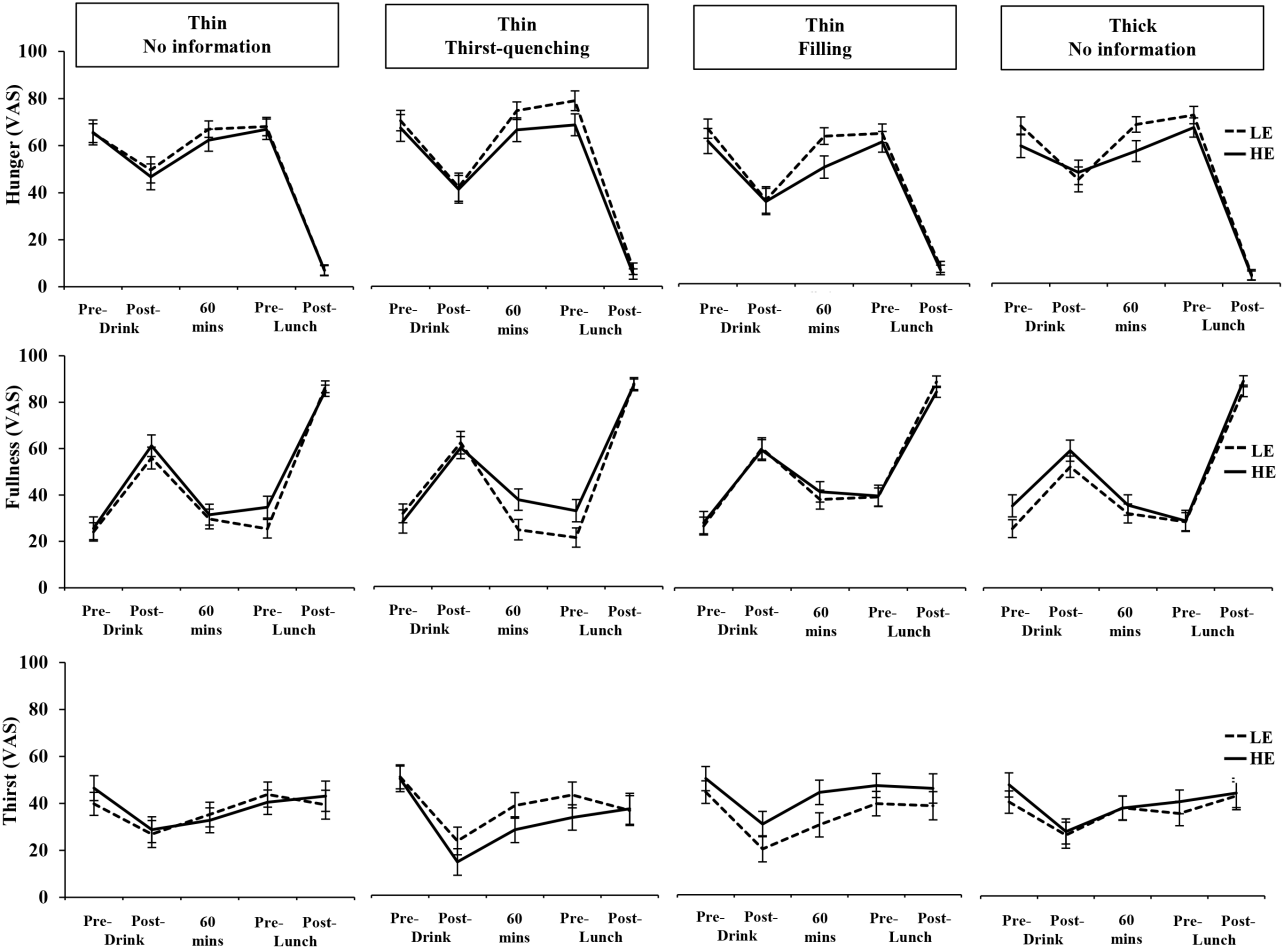
Hunger, fullness and thirst VAS ratings pre-drink, post-drink, 60 minutes later, pre-lunch and post-lunch, across each of the drink context groups.

Changes in hunger, fullness and thirst ratings throughout the test days are presented in Figure 4. Rated hunger decreased immediately after consuming all drinks, increasing back towards original levels before lunch, and decreasing again after lunch was consumed (*F*(3,244) = 325.51, *p*<0.001, *η_p_^2^* = 0.82). The reverse was seen with fullness ratings (*F*(4,256) = 342.76, *p*<0.001, *η_p_^2^* = 0.83). Furthermore, changes in rated hunger over time depended on the energy content of the beverage (*F*(3,239) = 3.31, *p* = 0.016, *η_p_^2^* = 0.05) as the HE drinks suppressed hunger more than the LE drinks in the interval between consuming the drink and eating the pasta. This was the same across the four beverage contexts (*F*(10,239) = 062, *p* = 0.798, *η_p_^2^* = 0.03). There was also a trend for the HE drinks to increase fullness in the period before lunch more than the LE drinks, which was primarily for those consuming the drinks in the thin/thirst-quenching beverage context (*F*(12,278) = 1.68, *p* = 0.072, *η_p_^2^* = 0.07; see Figure 4). The main effect of drink condition on hunger and fullness ratings and all other interactions were non-significant (*p*≥0.134, *η_p_^2^*≤0.06). Ratings of thirst also changed over time (*F*(3,241) = 19.80, *p*<0.001, *η_p_^2^* = 0.22): thirst decreased immediately after consuming the drink, and then increased over the next 60 minutes. There was no significant effect of drink energy on thirst ratings (*F*(1,70) = 1.27, *p* = 0.264, *η_p_^2^* = 0.02) nor did the drink’s energy content influence changes in thirst over time (*F*(4,259) = 0.66, *p* = 0.612, *η_p_^2^* = 0.01). However, there was evidence that the beverage context interacted with beverage energy content to influence thirst ratings overall (*F*(3,70) = 3.28, *p* = 0.026, *η_p_^2^* = 0.12), with participants in the *thin/filling* groups reporting being more thirsty on the HE day compared to the LE day (*p* = 0.007) whereas there was a trend for the opposite in the *thin/thirst-quenching* group (*p* = 0.098). Participants in the thin and thick *no information* groups reported being similarly thirsty across the HE and LE drinks days (*p*≥0.362). No other effects or interactions were significant (*p*≥0.612, *η_p_^2^*≤0.03).

### Water-intake

The amount of water participants consumed during the test days differed depending on the time of day (*F*(2,140) = 53.39, *p*<0.001, *η_p_^2^* = 0.43): participants tended to consume slightly less during lunch (*M* = 201.6±11.3 g) than in the 3 hour gap between breakfast and the test drink (*M* = 237.9±13.5 g, *p* = 0.052), and the least in the 60 minute gap between the drink and lunch (*M* = 99.3±10.9 g; *p*>0.001 for both comparisons). There was no evidence that water intake after consuming the test drink was different depending on the beverage context (*F*(6,140) = 0.90, *p* = 0.496, *η_p_^2^* = 0.04) or energy content (*F*(2,129) = 0.698, *p* = 0.488, *η_p_^2^* = 0.01). There were no other interactions or main effects (*p*≥0.440, *η_p_^2^*≤0.04).

### Sensory Ratings of the Test Products

The drinks were designed so that the thick LE and HE versions (used in the *thick/no-information* context) were perceived to be thicker and creamier than the thin LE and HE versions (used in the three thin contexts: *no information*, *thirst-quenching* and *filling*). The mean sensory and hedonic ratings for the tests drinks are presented in Table 3. Perceived thickness did differ across the four beverage contexts (*F*(3,70) = 3.34, *p* = 0.024, *η_p_^2^* = 0.13): the thick drinks were rated as thicker than the thin drinks consumed in the *thirst-quenching* context but not compared to the thin drinks consumed in the *no information* or *filling* contexts. While there was no overall effect of energy content on rated thickness (*F*(1,70) = 0.05, *p* = 0.818, *η_p_^2^*<0.01), a beverage context by energy content interaction suggested that the HE drinks were rated as subtly thicker than the LE versions in the thin/thirst-quenching group, but not in any other beverage context (*F*(3,70) = 3.18, *p* = 0.029, *η_p_^2^* = 0.12; see Table 2). Rated creaminess also differed between beverage context groups (*F*(3,70) = 3.75, *p* = 0.015, *η_p_^2^* = 0.14) following the same pattern as the thickness ratings: the thicker drinks were rated as creamier than the thin drinks consumed in the *thirst-quenching* context only. Beverage context did not interact with energy content to influence creaminess ratings (*F*(3,70) = 0.28, *p* = 0.838, *η_p_^2^* = 0.01), but there was an overall effect of energy content (*F*(1,70) = 5.43, *p* = 0.023, *η_p_^2^* = 0.07) with the LE drinks rated as slightly creamier than the HE versions (see Table 3). As for the rated pleasantness of the drinks, this depended on the beverage context and energy content (*F*(3,56) = 3.47, *p* = 0.021, *η_p_^2^* = 0.13): although both versions were rated highly, the thick LE drink was rated as more pleasant than the thick HE drink while there was a trend for the opposite in the thin/thirst-quenching group (see Table 3). The LE and HE versions did not differ in rated pleasantness in any of the other thin beverage context groups. There was no main effect of beverage context or energy content on rated pleasantness (*p*≥0.384, *η_p_^2^*≤0.04 for both main effects). Otherwise, the drinks were all rated as similarly sweet and familiar (for all main effects and interactions *p*≥0.356, *η_p_^2^*≤0.05).

**Table 3:** Mean (±SEM) sensory ratings of the higher- and lower- energy test drinks in each beverage context condition.

		Thin	Thin	Thin	Thick
		No information	Thirst-quenching	Filling	No information
Thick^a^	LE	67.2±4.0	54.9±4.2	63.4±4.0	75.3±4.0
	HE	67.1±4.2	63.2±4.5	60.7±4.2	73.3±4.2
Creamy^b^	LE	66.1±3.9	60.5±4.1	67.6±3.9	76.5±3.9
	HE	60.5±4.6	52.7±4.9	65.4±4.6	72.1±4.6
Sweet^c^	LE	74.1±3.0	73.4±3.2	73.4±3.0	71.2±3.0
	HE	76.6±2.9	70.4±3.2	71.4±2.9	73.1±2.9
Familiar^c^	LE	68.5±5.1	64.9±5.4	63.8±5.1	67.1±5.1
	HE	75.2±5.3	58.4±5.6	67.7±5.3	71.5±5.3
Pleasant^d^	LE	74.7±3.4	80.9±3.6	81.9±3.4	81.8±3.4
	LE	78.6±4.0	87.4±4.2	82.0±4.0	74.2±4.0

a Overall the *thick* drinks were rated as thicker than the thin drinks consumed in the *thirst-quenching* context (*p* = 0.028) but not compared to the thin drinks consumed in the *no information (p = *0.797*)* or *filling* contexts (*p* = 0.101). All of the thin drinks were rated as similarly thick (*p*≥0.919). The beverage context by energy content interaction indicated that the HE beverage was rated as subtly thicker than the LE version in the *thirst-quenching* group *(p* = 0.011) but thickness ratings for the LE and HE beverages did not differ in any other groups (*p*≥0.177).

b The *thick* drinks were rated as thicker than the thin drinks consumed in the *thirst-quenching* context (*p* = 0.010) but not compared to the thin drinks consumed in the *no information (p = *0.233*)* or *filling* contexts (*p* = 0.841). All of the thin drinks were rated as similarly thick (*p*≥0.426). Overall, there was a main effect of energy content indicating that the LE beverages (*M* = 67.7±2.0) were rated as creamier than the HE beverages (*M* = 62.7±2.3; *p* = 0.023).

c Ratings of sweetness and familiarity did not differ across beverage contexts or energy contents.

d The beverage context by energy content interaction indicated that in the *thick/no information* group the HE beverage were rated as less pleasant than the LE version. Pleasantness ratings for the LE and HE beverages did not differ in any other groups (*p*≥0.238), although there was a trend for the LE beverages to be rated as slightly less pleasant than the HE versions (*p*≥0.065) in the *thin/thirst-quenching.*

Participants rated the pasta lunch as similarly pleasant across the test sessions and these ratings did not depend on the beverage context or energy content (*p*≥0.155, *η_p_^2^*≤0.07 for each main effect and the interaction). The pasta lunch was also rated as similarly familiar and salty (*p*≥0.102, *η_p_^2^*≤0.08 for all main effects and interactions).

### Debriefing Questionnaire

No participant correctly identified the purpose of the study to be investigating the role of beverage context on satiety responses to a covert manipulation of a beverage’s energy content. Participants’ beliefs about the purpose of the study depended on whether they were given extra information about the beverage. In line with the general study cover story, the majority of participants who did not receive explicit information about the drink products (both no information groups) reported that the purpose of the study was to investigate “food and mood” (61%) with the remaining participants making general suggestions such as “food and appetite” “snacking” and “food behaviour”. The majority of participants who received information that the drink was designed to be either thirst-quenching or filling reported the effect of the drink on “mood”, “fullness” and/or “thirst” as the purpose of the study (69%), in line with what they were told, with the remaining participants reporting other things such as “product testing”, “overeating” and “food planning”. Crucially, no one identified that the drinks differed in energy content. Overall, 69% of participants believed that the drinks they consumed were the same on both days, 11% reported that they “didn’t know” whether the drinks were different and 19% identified that the drinks were different because one drink had a different “taste” or one was more “enjoyable” than another. Two of these participants believed that one drink was more filling than the other but did not suggest why.

Table 4 outlines participants’ expectations about the drinks. Most participants in the no information groups reported that they expected the drinks to be filling. Most participants who were told the drinks would be thirst-quenching reported expecting them to be “thirst-quenching”, while participants who consumed the beverage as a filling snack expected the drinks to be ‘filling’ and “both thirst-quenching and filling”.

**Table 4:** The reported expectations of the test drinks, recorded during the debrief session.

	Thin	Thin	Thin	Thick
	No information	Thirst-quenching	Filling	No information
Thirst-quenching	2	10	0	2
Filling	8	1	9	10
Both	6	6	10	6
Neither	3	0	0	1
Other	0	0	0	0
*n* =	19	17	19	19

## Discussion

The findings from this study indicate that the cognitive and sensory context in which a beverage is consumed can influence the satiety value it affords the consumer. Participants who consumed thin beverages without any extra contextual information showed a weak satiety response to the additional 197 kcal in the higher-energy test drink, eating the same amount of lunch after both the lower- and higher-energy versions of the beverage. A similar effect was seen in participants who were led to believe that the drinks were designed to be thirst-quenching. However, when the same beverages were presented as a filling snack, participants responded to the additional energy in the higher-energy beverage by adjusting their intake at a later lunch. This effect was also seen in participants who consumed the subtly thicker beverages, who showed the largest adjustment to lunch intake after consuming the higher- and lower-energy beverages. This indicates that for a beverage containing a substantial amount of energy, encouraging people to consider it a snack that will affect hunger and fullness, rather than just a drink, could influence its satiating power. This offers an alternative strategy to modifying a beverage’s sensory profile, which is likely to be unacceptable to consumers of many popular low-viscosity but higher-energy beverages such as flavoured waters, soft drinks, sports beverages and energy drinks.

The idea that the context of consumption affects the satiating power of nutrients is consistent with the view that early pre-consumption signals (sensory experience, environmental cues, beliefs and memories about the consequences of consuming a food or drink) integrate with later post-ingestive and post-absorptive feedback from nutrients to determine satiety [16], [17]. When a food is believed to be satiating these thoughts about the consequences of consuming a product can have large effects on the physiological response to food, such as eliciting slower gastro-intestinal transit time and a larger decline in levels of the orexigenic hormone ghrelin post-ingestion [7]. The early cognitive and sensory signals generated by food and drinks are thought to enhance satiety by priming the appetite system for the delivery of nutrients [41], [42]. For many low-viscosity energy-containing beverages that are consumed fast and as a drink, the cognitive and sensory cues may not be strong enough to elicit such preparatory responses. Subtle thick and creamy sensory cues can increase the expectation that a beverage will be more satiating than the same beverage without these cues [23], and these sensory modifications (which did not add any energy to the beverages) can also improve the actual satiating power of a higher-energy beverage when it was consumed [26]–[28]. The current study extends this to show that making the context of consuming an energy-containing beverage more satiety-relevant by changing consumer beliefs alone may also influence its satiety value, but to a lesser extent.

However, in this study early cognitive and sensory cues did not have a general effect on satiety (there was no overall effect of beverage context on lunch intake). This suggests that the satiety-relevant cues did not consistently enhance the satiety value of both the higher- and lower-energy beverages. A potential consequence of an appetite system primed for nutrients is that if the post-ingestive nutrient effects were less than anticipated, a person might actually experience less satiety than if the satiety-relevant cues were absent in the first place. Yeomans and Chambers [27] reported some preliminary evidence for this effect, which they termed ‘rebound hunger’. They found that making a higher-energy beverage thicker and creamier resulted in reduced intake at a subsequent lunch, however when the same sensory manipulations were applied to a lower-energy version of the beverage participants reported increased hunger and tended to eat more at lunch compared to when they had consumed the same lower-energy drink without these sensory enhancements. Thus, the differences in lunch intake reported in this study could have been due to a decrease in intake after the higher-energy beverage (enhanced satiety), an increase in intake after the lower-energy beverage (rebound hunger), or a combination of both. The participants consuming the beverages in the thick and filling context groups demonstrated the largest response to the beverages energy content, but they also tended to eat the most after the lower-energy beverages. This suggests satiety may have been reduced after the lower-energy beverage when it was presented in a satiety-relevant context, although the appetite ratings do not supported this. Directly testing the combination of satiety-relevant cues and energy levels in foods and beverages that combine to enhance satiety or induce rebound hunger will be an important consideration for future research.

Although explicit beliefs about the consequences of consuming a food can impact the actual satiety value of a food or drink, in the present study changing the sensory rather than cognitive context of beverage consumption had the greatest impact on satiety responses to the additional energy, perhaps because food texture is a strong predictive cue for the presence of nutrients [42], whereas received information (particularly in a laboratory context) may be a less reliable source of information. An alternative explanation for the satiating effect of the thicker higher-energy beverage in this study is that the thickening agent, tara gum, had a post-ingestive effect on satiety. While there is evidence to suggest that consuming similar polysaccharide thickeners (such as guar gum) can reduce appetite, this effect is small and requires much larger quantities of fibre (e.g. ≈10 g [43]) per serving than the 1.0 g serving used in the present study. Furthermore, the addition of 1.2 g of tara gum to the low-energy thick beverage did not enhance its satiating power in this study nor in our previous research [26]–[28], an effect you would expect to see if the tara gum was having an independent effect on satiety. One possibility is that the thickener interacted with the additional energy in the higher energy beverage, perhaps by slowing the digestion of these extra nutrients, but this is unlikely given the small quantity of tara gum used and its subtle effect on viscosity. In the present study consuming higher- and lower-energy beverages in the context of a filling snack also influenced their satiating power, whereas the same drinks consumed with either no information or the belief that it would be thirst-quenching did not elicit different effects on satiety, despite having the same energy difference and the same viscosity as those consumed as a filling snack. Thus, it is plausible that the thicker beverages influenced the satiating power of the additional nutrients through changing their anticipated satiety value rather than an independent post-ingestive effect of the thickener alone.

Despite intake at lunch after the higher- and lower-energy beverages depending on the beverage context, ratings of hunger and fullness did not. Participants reported feeling more full and less hungry after consuming the higher-energy compared to the lower-energy version, indicating that the rating scale used to make these judgements was sensitive to appetite changes. Research suggests that ratings of appetite alone are not always accurate predictors of energy intake at a next meal due to their subjective nature and variation in the way they are expressed by different individuals [44]–[46]. This may help to explain why differences in ratings of appetite were only apparent for the within-participant manipulation of the drink’s energy content. Perceived sensations of thirst were not affected by any of the beverage characteristics (cognitive or sensory context, or energy content). This is not necessarily surprising because ratings of thirst and motivations to drink are thought to be relatively high and consistent throughout the day [45], [47], and participants did consume similar amounts of water across the test days.

A limitation of the study is that we did not formally assess what participants’ expected from the beverages as they were consuming them, relying instead on debrief reports once the study was complete. As this study was conducted in a laboratory it was anticipated that demand effects would heavily influence a measure of expectations taken at the point of consumption, particularly for participants who received explicit information about the beverages. This could have affected later lunch intake if participants felt that they had to eat in accordance with their rated expectations. However, measuring expectations retrospectively as we did may have provided a less accurate report of each participant’s true expectations. Nevertheless, the debrief data did suggest that the participants expected the drinks to be more filling and thirst-quenching in accordance with the information they received, and as the main findings of the study were in line with the prediction (that participants would be better able to respond to the energy content of the beverage when they were consumed in a context more consistent with satiety) this indicates that the cognitive manipulations were successful for the most part.

It was unexpected that the sensory evaluations of the test drinks would be influenced by our cognitive manipulations. The thicker beverages were rated thicker and creamier than the thin versions only when consumed in the thirst-quenching context, even though participants in the other two beverage context groups (*filling snack* and *no information*) consumed the same thin beverages. In our previous research these subtle textural manipulations were highly perceptible when thick and thin versions were compared side-by-side in a taste test and the higher- and lower-energy versions were well matched [23]. In the present study participants consumed either thin or thick versions of the beverages, so differences in perceived thickness and creaminess were probably less evident between beverage context groups as they were when compared by the same person. Importantly, the higher- and lower-energy beverages were fairly well matched for sensory characteristics and participants were not aware of the energy manipulation within the test-drinks.

Overall, data from this study indicates that changing certain features of energy-containing beverages can influence the effect it has on the amount of food subsequently consumed: changing the context in which a beverage is consumed from a *drink* to a *snack* impacts a person’s satiety response to the energy it contains, although the most effective strategy was to change its sensory characteristics to be more predictive of nutrients. These data represent short-term influences on eating behaviour within a laboratory environment where food intake was controlled. To move forward, it is important to consider whether consuming energy-containing beverages in a more satiety-relevant context will influence satiety responses outside of a laboratory setting, when a person not only decides how much of a food they consume but also what and when they eat or drink. Encouragingly, contextual cues from a products marketing, labelling, presentation and sensory profile can influence eating behaviour in real-world settings such as in restaurants, supermarkets and at home [48]. Energy rich meal-replacement beverages can have a positive impact on intake regulation and even promote weight loss when consumed in the context of “food” [32], albeit in people committed to losing weight. Future research should focus on appropriate ways to promote liquid calories as fuel rather than fluid and to determine the impact of this approach over the longer term on product selection and energy intake.

## References

[1] Popkin BM, Adair LS, Ng SW (2012) Global nutrition transition and the pandemic of obesity in developing countries. Nutr Rev 70: 3–21. 22221213 10.1111/j.1753-4887.2011.00456.x PMC3257829

[2] Van Kleef E, Van Trijp JCM, Van den Borne J, Zondervan C (2012) Successful Development of Satiety Enhancing Food Products: Towards a Multidisciplinary Agenda of Research Challenges. Crit Rev Food Sci Nutr 52: 611–628. 22530713 10.1080/10408398.2010.504901 PMC3662086

[3] Almiron-Roig E, Palla L, Guest K, Ricchiuti C, Vint N, et al (2013) Factors that determine energy compensation: a systematic review of preload studies. Nutr Rev 71: 458–473. 23815144 10.1111/nure.12048 PMC3746122

[4] DellaValle DM, Roe LS, Rolls BJ (2005) Does the consumption of caloric and non-caloric beverages with a meal affect energy intake? Appetite 44: 187–193. 15808893 10.1016/j.appet.2004.11.003

[5] Panahi S, El Khoury D, Luhovyy BL, Goff HD, Anderson GH (2013) Caloric beverages consumed freely at meal-time add calories to an ad libitum meal. Appetite 65: 75–82. 23402713 10.1016/j.appet.2013.01.023

[6] Rolls BJ, Kim S, Fedoroff IC (1990) Effects of drinks sweetened with sucrose or aspartame on hunger, thirst and food-intake in men. Physiol Behav 48: 19–26. 2236270 10.1016/0031-9384(90)90254-2

[7] Cassady BA, Considine RV, Mattes RD (2012) Beverage consumption, appetite, and energy intake: what did you expect? Am J Clin Nutr 95: 587–593. 22258267 10.3945/ajcn.111.025437 PMC3278240

[8] Mattes RD, Campbell WW (2009) Effects of Food Form and Timing of Ingestion on Appetite and Energy Intake in Lean Young Adults and in Young Adults with Obesity. J Am Diet Assoc 109: 430–437. 19248858 10.1016/j.jada.2008.11.031 PMC2680008

[9] Mourao DM, Bressan J, Campbell WW, Mattes RD (2007) Effects of food form on appetite and energy intake in lean and obese young adults. Int J Obes 31: 1688–1695. 10.1038/sj.ijo.0803667 17579632

[10] Tournier A, Louis-Sylvestre J (1991) Effect of the physical state of a food on subsequent intake in human subjects. Appetite 16: 17–24. 2018401 10.1016/0195-6663(91)90107-4

[11] Cabrera Escobar M, Veerman J, Tollman S, Bertram M, Hofman K (2013) Evidence that a tax on sugar sweetened beverages reduces the obesity rate: a meta-analysis. BMC Public Health 13: 1072. 24225016 10.1186/1471-2458-13-1072 PMC3840583

[12] Fletcher JM, Frisvold D, Tefft N (2010) Can soft drink taxes reduce population weight? Contemp Econ Policy 28: 23–35. 20657817 10.1111/j.1465-7287.2009.00182.x PMC2908024

[13] Holt E (2011) Hungary to introduce broad range of fat taxes. The Lancet 378: 755. 10.1016/s0140-6736(11)61359-7 21877327

[14] Kleiman S, Ng SW, Popkin B (2012) Drinking to our health: can beverage companies cut calories while maintaining profits? Obes Rev 13: 258–274. 22070346 10.1111/j.1467-789X.2011.00949.x PMC3420345

[15] Ng SW, Mhurchu CN, Jebb S, Popkin BM (2012) Pattern and trends of beverage consumption among children and adults in Great Britain, 1986–2009. Br J Nutr 108: 536–551. 22186747 10.1017/S0007114511006465 PMC3310974

[16] Blundell JE, de Graaf C, Hulshof T, Jebb S, Livingstone BM, et al (2010) Appetite control: methodological aspects of the evaluation of foods. Obes Rev 11: 251–270. 20122136 10.1111/j.1467-789X.2010.00714.x PMC3609405

[17] Blundell JE, Rogers PJ, Hill AJ, editors (1987) Evaluating the satiating power of foods. Implications for acceptance and consumption: In Colms, J., Booth, D.A., Pangborn, R.M. & Raunhardt, O. (Eds.). *Food acceptance and nutrition*. Academic Press: London, 1987, 205–219.

[18] Mattes RD (2006) Beverages and positive energy balance: the menace is the medium. Int J Obes 30: S60–S65.

[19] Hogenkamp PS, Mars M, Stafleu A, de Graaf C (2012) Repeated consumption of a large volume of liquid and semi-solid foods increases ad libitum intake, but does not change expected satiety. Appetite 59: 419–424. 22721908 10.1016/j.appet.2012.06.008

[20] Zhu Y, Hsu WH, Hollis JH (2013) The Impact of Food Viscosity on Eating Rate, Subjective Appetite, Glycemic Response and Gastric Emptying Rate. PloS ONE 8: e67482. 23818981 10.1371/journal.pone.0067482 PMC3688614

[21] Zijlstra N, Mars M, de Wijk RA, Westerterp-Plantenga MS, de Graaf C (2008) The effect of viscosity on ad libitum food intake. Int J Obes 32: 676–683. 10.1038/sj.ijo.0803776 18071342

[22] Hogenkamp PS, Stafleu A, Mars M, Brunstrom JM, de Graaf C (2011) Texture, not flavor, determines expected satiation of dairy products. Appetite 57: 635–641. 21871509 10.1016/j.appet.2011.08.008

[23] McCrickerd K, Chambers L, Brunstrom JM, Yeomans MR (2012) Subtle changes in the flavour and texture of a drink enhance expectations of satiety. Flavour 1.

[24] Woods SC (2009) The Control of Food Intake: Behavioral versus Molecular Perspectives. Cell Metab 9: 489–498. 19490904 10.1016/j.cmet.2009.04.007 PMC3090647

[25] Bertenshaw EJ, Lluch A, Yeomans MR (2013) Perceived thickness and creaminess modulates the short-term satiating effects of high-protein drinks. Br J Nutr 110: 578–586. 23312079 10.1017/S0007114512005375

[26] Chambers L, Ells H, Yeomans MR (2013) Can the satiating power of a high energy beverage be improved by manipulating sensory characteristics and label information? Food Qual Prefer 28: 271–278.

[27] Yeomans MR, Chambers L (2011) Satiety-relevant sensory qualities enhance the satiating effects of mixed carbohydrate-protein preloads. Am J Clin Nutr 94: 1410–1417. 22030223 10.3945/ajcn.111.011650

[28] Yeomans MR, McCrickerd K, Brunstrom JM, Chambers L (2014) Effects of repeated consumption on sensory-enhanced satiety. Br J Nutr 111: 1137–1144. 24229713 10.1017/S0007114513003474

[29] Mattes RD (2005) Soup and satiety. Physiol Behav 83: 739–747. 15639159 10.1016/j.physbeh.2004.09.021

[30] Hogenkamp PS, Mars M, Stafleu A, de Graaf C (2010) Intake during repeated exposure to low- and high-energy-dense yogurts by different means of consumption. Am J Clin Nutr 91: 841–847. 20164319 10.3945/ajcn.2009.28360

[31] Wansink B, Payne CR, Shimizu M (2010) “Is this a meal or snack?” Situational cues that drive perceptions. Appetite 54: 214–216. 19808071 10.1016/j.appet.2009.09.016

[32] Heymsfield SB, van Mierlo CAJ, van der Knaap HCM, Heo M, Frier HI (2003) Weight management using a meal replacement strategy: meta and pooling analysis from six studies. Int J Obes 27: 537–549. 10.1038/sj.ijo.0802258 12704397

[33] Capaldi ED, Owens JQ, Privitera GJ (2006) Isocaloric meal and snack foods differentially affect eating behavior. Appetite 46: 117–123. 16442668 10.1016/j.appet.2005.10.008

[34] Martens MJI, Westerterp-Plantenga MS (2012) Mode of consumption plays a role in alleviating hunger and thirst. Obesity (Silver Spring) 20: 517–524. 22095116 10.1038/oby.2011.345

[35] Pliner P, Zec D (2007) Meal schemas during a preload decrease subsequent eating. Appetite 48: 278–288. 17250926 10.1016/j.appet.2006.04.009

[36] Brunstrom JM, Brown S, Hinton EC, Rogers PJ, Fay SH (2011) ‘Expected satiety’ changes hunger and fullness in the inter-meal interval. Appetite 56: 310–315. 21219951 10.1016/j.appet.2011.01.002

[37] Stunkard AJ, Messick S (1985) The 3-Factor Eating Questionnaire to Measure Dietary Restraint, Disinhibition and Hunger. J Psychosomat Res 29: 71–83. 10.1016/0022-3999(85)90010-8 3981480

[38] Koliandris AL, Morris C, Hewson L, Hort J, Taylor AJ, et al (2010) Correlation between saltiness perception and shear flow behaviour for viscous solutions. Food Hydrocoll 24: 792–799.

[39] Yeomans MR (2000) Rating changes over the course of meals: what do they tell us about motivation to eat? Neurosci Biobehav Rev 24: 249–259. 10714388 10.1016/s0149-7634(99)00078-0

[40] Cohen J (1988) Statistical power analysis for the behavioral sciences. Hillsdale, NJ: Lawrence Earlbaum Associates.

[41] Brunstrom JM (2007) Associative learning and the control of human dietary behavior. Appetite 49: 268–271. 17197053 10.1016/j.appet.2006.11.007

[42] Davidson TL, Swithers SE (2004) A Pavlovian approach to the problem of obesity. Int J Obes 28: 933–935. 10.1038/sj.ijo.0802660 15111986

[43] Wanders AJ, van den Borne J, de Graaf C, Hulshof T, Jonathan MC, et al (2011) Effects of dietary fibre on subjective appetite, energy intake and body weight: a systematic review of randomized controlled trials. Obes Rev 12: 724–739. 21676152 10.1111/j.1467-789X.2011.00895.x

[44] Mattes RD (1990) Hunger ratings are not a valid proxy measure of reported food-intake in humans. Appetite 15: 103–113. 2268136 10.1016/0195-6663(90)90043-8

[45] Mattes RD (2010) Hunger and thirst: Issues in measurement and prediction of eating and drinking. Physiol Behav 100: 22–32. 20060847 10.1016/j.physbeh.2009.12.026 PMC2849909

[46] Stubbs RJ, Hughes DA, Johnstone AM, Rowley E, Reid C, et al (2000) The use of visual analogue scales to assess motivation to eat in human subjects: a review of their reliability and validity with an evaluation of new hand-held computerized systems for temporal tracking of appetite ratings. Br J Nutr 84: 405–415. 11103211 10.1017/s0007114500001719

[47] McKiernan F, Houchins JA, Mattes RD (2008) Relationships between human thirst, hunger, drinking, and feeding. Physiol Behav 94: 700–708. 18499200 10.1016/j.physbeh.2008.04.007 PMC2467458

[48] Cohen DA, Babey SH (2012) Contextual influences on eating behaviours: heuristic processing and dietary choices. Obes Rev 13: 766–779. 22551473 10.1111/j.1467-789X.2012.01001.x PMC3667220

